# SOD1, an unexpected novel target for cancer therapy

**DOI:** 10.18632/genesandcancer.4

**Published:** 2014-01

**Authors:** Luena Papa, Giovanni Manfredi, Doris Germain

**Affiliations:** ^1^ From the Department of Medicine, Division of Hematology/Oncology, Tisch Cancer Institute Mount Sinai School of Medicine, One Gustave L. Levy Place, New York, NY; ^2^ The Weill Cornell Medical College, Department of Neurology and Neuroscience, New York, New York

**Keywords:** SOD1, SOD2, SIRT3, cancer, fALS

## Abstract

Cancer cells have elevated levels of reactive oxygen species (ROS), which are generated in majority by the mitochondria. In the mitochondrial matrix, the manganese dismutase SOD2 acts as a major anti-oxidant enzyme. The deacetylase SIRT3 regulates the activity of SOD2. Recently, SIRT3 was reported to be decreased in 87% of breast cancers, resulting therefore in a decrease in the activity of SOD2 and an elevation in ROS. In addition to SIRT3, we recently reported that SOD2 itself is down-regulated in breast cancer cell lines upon activation of oncogenes, such as Ras. Since in absence of SOD2, superoxide levels are elevated and may cause irreversible damage, mechanisms must exist to retain superoxide below a critical threshold and maintain viability of cancer cells. The copper/zinc dismutase SOD1 localizes in the cytoplasm, the inter-membrane space of the mitochondria and the nucleus. Emerging evidences from several groups now indicate that SOD1 is overexpressed in cancers and that the activity of SOD1 may be essential to maintain cellular ROS under this critical threshold. This review summarizes the studies reporting important roles of SOD1 in cancer and addresses the potential cross-talk between the overexpression of SOD1 and the regulation of the mitochondrial unfolded protein response (UPR^mt^). While mutations in SOD1 is the cause of 20% of cases of familial amyotrophic lateral sclerosis (fALS), a devastating neurodegenerative disease, these new studies expand the role of SOD1 to cancer.

## INTRODUCTION

The copper/zinc dismutase SOD1 is an abundant enzyme required for the conversion of superoxide to hydrogen peroxide. SOD1 localizes mainly to the cytoplasm but is also found in the nucleus and the inter-membrane space (IMS) of the mitochondria. In familial amyotrophic lateral sclerosis (fALS), the IMS-fraction of SOD1, although a minor fraction of total cellular SOD1, appears to play an important role. This review focuses on an emerging role of SOD1 in cancer biology, where as in ALS, we propose that the IMS-fraction may be of significant importance by playing dismutase-dependent and independent roles.

### The IMS of the mitochondria

Mitochondria are comprised of the matrix, the inner-membrane, the outer-membrane, and the space between the inner-membrane and the outer-membrane, referred to as the inter-membrane space (IMS). Contrary to the intensive studies of the inner-membrane (electron transport chain), outer-membrane (permeabilization during apoptosis, fusion/fission), and the matrix (Kreb cycle, amino acid metabolism, etc.), the IMS has been largely overlooked. The general view is that the IMS is a passive sub-compartment, which acts mainly as a storage space of pro-apoptotic proteins until they are needed for the execution of cell death. However, this view is far from reality.

The IMS contains over 100 proteins [1]. Collectively, the various functions of these proteins indicate that the IMS acts as a logistic hub that orchestrates metabolic processes, import of proteins, oxidative folding, protein degradation, transport of metabolites, lipids and metals ions, export of ferrous precursors, assembly of the respiratory chain, detoxification of reactive oxygen species (ROS) and ROS-mediated signaling [1]. Therefore, defects in the function of the IMS impact the entire organelle.

The activity of the electron transport chain is the main source of ROS in the IMS. However in addition, oxidative protein folding also leads to ROS accumulation in the IMS. The IMS is only one of the two cellular compartments, where this process takes place; the other is the endoplasmic reticulum. Each cycle of folding generates one molecule of ROS. Oxidation of cysteins leads to formation of disulfide bonds, which if inappropriate, can lead to misfolding and protein aggregation. Therefore, the IMS can be considered as a highly oxidative cellular sub-compartment, implying that proteins in the IMS maybe at high risk of misfolding [1]. Since SOD1 localizes to the IMS, its dismutase activity is likely to be essential to limit the accumulation of ROS and misfolded proteins in this sub-compartment of the mitochondria but this possibility remains to be formally tested. The importance of SOD1 in the IMS is best illustrated by the role of SOD1 in fALS.

### SOD1-G93A in familial ALS (fALS) and the involvement of IMS-stress

Mutations in SOD1 are responsible for approximately 20% of fALS [2]. The pathophysiology of SOD1-ALS is not completely understood, and different mechanisms may participate in pathogenesis [3], including mitochondrial dysfunction [4, 5]. SOD1-ALS is a non-cell autonomous disease, meaning that the mutant protein has a toxic effect in multiple cell types, including neurons and glia, and that these effects are additive [6]. The toxic role of SOD1 mutant astrocytes for motor neurons is well documented, both in vitro [7] and in vivo [8], and importantly it has been confirmed also in astrocytes derived from sporadic ALS patients [9]. The G93A amino acid substitution in SOD1 is the one of the most extensively studied mutations, both in cultured cells and in mouse models of the disease. Transgenic mice express high levels of SOD1-G93A ubiquitously, under the control of the SOD1 endogenous promoter. They rapidly develop motor neuron degeneration, resulting in paralysis and death by 4 or 5 months of age, depending on the genetic background [10]. The prevalent theory for the pathogenesis of mutant SOD1 involves a gain of toxic function of SOD1-G93A. Mutant SOD1 has pleiotropic effects in cells: for example, organelles, such as endoplasmic reticulum [11], mitochondria, and peroxisomes [12] present distinct abnormalities in SOD1 mutant motor neurons. Mutant SOD1 affects the integrity of the neuronal cytoskeleton [13] [14] possibly impairing the support of normal trafficking along axons and dendrites. Mutant SOD1 alters intracellular signaling [15], decreases protein quality control activity [16], activates cell death pathways [17] [18], promotes aberrant free radical production [19], and decreases the levels of crucial receptors, such as Glur2 [20] and transporters such as the astrocytic glutamate transporter [21]. Mutant SOD1 is also secreted outside cells, where it may induce neuroinflammatory responses [22]. Several significant findings on the involvement of mitochondria in fALS have derived from the work of several groups in SOD1-G93A models. One critical point for this review is that misfolded SOD1 localizes to multiple cell compartments, including mitochondria. Mutant SOD1 accumulates on the mitochondrial outer membrane [23] where it interacts with some crucial proteins, such as Bcl2 [24] and VDAC [25]. However, mutant SOD1 also localizes inside the mitochondrial IMS, where it accumulates and misfolds, potentially interfering with the assembly and maturation of mitochondrial proteins [26-28]. The pathogenic role of the IMS pool of mutant SOD1 is supported by evidence form cultured motorneuronal cells, where it causes mitochondrial functional, morphological, and axonal transport abnormalities [29, 30]. Recently, transgenic mice expressing SOD1-G93A in the IMS but not the cytoplasm [IMS-only SOD1-G93A] were generated and found to develop some of the symptoms of ALS, including motor defects, and mitochondrial abnormalities [31]. These findings demonstrate an important consequence of the accumulation of mutant SOD1 in the IMS.

Further support of the importance of the IMS-fraction of SOD1 arise from the finding that the peripheral neuropathy of the SOD1 knockout mice can be rescue by expression of wild-type SOD1 targeted specifically to the IMS [32]. This finding eloquently indicates that although considered a minor fraction relative to the cytoplasmic fraction, SOD1 in the IMS plays a crucial role in the integrity of the mitochondria. In addition to fALS, the importance of SOD1 is beginning to emerge as critical in cancer biology. While the contribution of SOD1 in the cytoplasm is undeniable, we propose the IMS-fraction of SOD1 may also play an important role in maintaining the viability of cancer cells and that SOD1 may potentially become a therapeutic target for cancer.

### The mitochondria and oxidative stress

The activity of the respiratory chain of the inner-membrane of the mitochondria generates reactive oxygen species (ROS), which can result in oxidative damage to mitochondrial DNA and proteins. Therefore, oxidative damage may be considered the major source of stress in this organelle.

**Fig 1:**
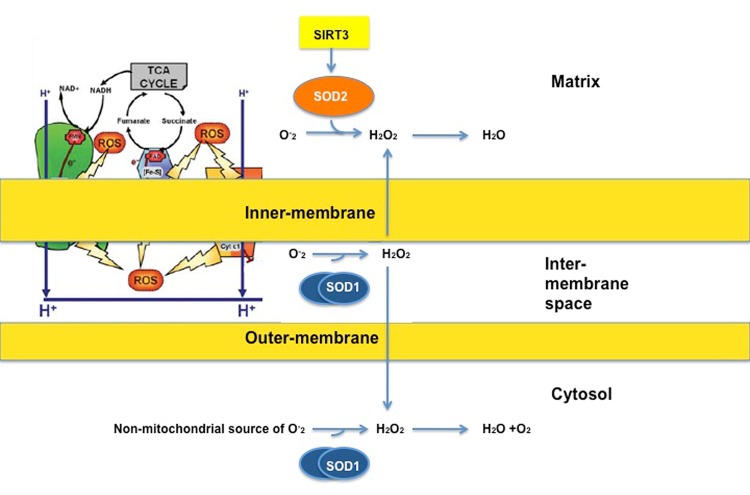
ROS is produced on both sides of the inner-membrane On the matrix side, the manganese dismutase SOD2 detoxifies superoxide (O-2) to hydrogen peroxide (H2O2), which is then being converted to water. SOD2 is activated by SIRT3. On the inter-membrane space side, superoxide is being converted to hydrogen peroxide by the copper/zinc dismutase SOD1. Hydrogen peroxide can then diffuse to the matrix or cytosol to be converted to water. Diagram taken and adapted from Lemarie et al, 2011.

ROS is produced on both sides of the inner-membrane of the respiratory chain (Fig. 1). The accumulation of ROS in the matrix is limited by the potent anti-oxidant machinery of this sub-compartment, where SIRT3 orchestrates the activity of the manganese superoxide dismutase SOD2 (Fig. 1). SOD2 in the matrix converts superoxide, which cannot diffuse across membranes, to hydrogen peroxide, which is diffusible (Fig. 1). In addition, the matrix contains enzymes to convert hydrogen peroxide to water. The anti-oxidant machinery of the matrix has been the focus of intensive research [33-35].

Compared to the matrix, the anti-oxidant machinery of the IMS is much more limited and relies on the activity of SOD1 (Fig. 1). Therefore the detoxification of superoxide by SOD1 appears to be essential in avoiding irreversible oxidative damage.

Adding to the importance of SOD1 in the mitochondria is a recent study from the Haigis' group showing that SIRT3 is either completely lost or reduced in 87% of breast cancers [36]. This work indicates that a decrease in SIRT3 may in fact be required for the metabolic reprogramming and the shift to glycolysis, which characterizes cancer cells [36, 37]. In addition the Gius' group have elegantly demonstrated that the deacetylase activity of SIRT3 is essential for the activity of SOD2. Therefore, the observation that in the absence of SIRT3, the activity of SOD2 is abolished [38], is a likely reason for the elevated ROS levels in cancer cells [39]. Moreover, mutations in the sub-units of the respiratory chain complex I, II and III were shown to lead to elevated production of superoxide in several cancers types (reviewed in, [40]). That cancer cells survive such elevated levels of superoxide is remarkable. Presumably, the AAA-proteases-mediated protein quality control and Lon protease [41] can limit the accumulation of dysfunctional proteins. Nevertheless, the stress imposed on the matrix remains considerably high. In this context, accumulation of ROS in the IMS would only add to the overall stress on the organelle and results in its collapse. Therefore, decreased expression of SIRT3 is likely to impose an immense demand on the anti-oxidant machinery of the IMS, namely SOD1.

### SOD1 is identified as a target in a small molecule inhibitors screen in lung cancer

The Varmus ‘s group has recently performed a high-throughput chemical screen to identify small molecule inhibitors of lung cancer cells. They reported the identification of LCS-1 as such a molecule, and subsequently combined affinity proteomics and gene expression analyses to identify the target of LCS-1 [42]. This led to the unexpected finding that SOD1 is the target of LCS-1 [42]. They reported in their study that overexpression of SOD1 promotes lung cancer cells growth and reduce apoptosis. Moreover, LCS-1 and its active analog, but not an inactive analog, inhibited the activity of SOD1. Further, they found that LCS-1 inhibits the growth of almost every cancer cell line in the 60 NIH set, including breast cancer lines.

In addition, Huang et al., identified SOD1 as a target of an anti-cancer agent in leukemia [43]. This data supports the hypothesis that SOD1 may be essential for the adaptation of cancer cells to elevated oxidative stress.

How inhibition of SOD1 leads to cancer cell death and whether inhibition of the IMS-fraction of SOD1 contributes to the effect of LCS-1 was not addressed. We recently reported that treatment of breast cancer cells with LCS-1 leads to a drastic alteration in the morphology of the mitochondria associated with increased fragmentation and swelling of the matrix [44]. This effect was not observed in the non-malignant breast epithelial cells line MCF10A [44]. Therefore, one interpretation of this finding is that inhibition of the IMS-fraction of SOD1, in cells where the expression of SIRT3 is low and the activity of SOD2 is compromised, may result in excessive mitochondrial damage and the collapse of the mitochondrial network, leading to cell death.

### Understanding the mechanism by which inhibition of SOD1 leads to cell death

A recent study by the Chandel's group further supports the notion of SOD1 as a target in cancer. They showed that inhibition of SOD1 either by shRNA or the SOD1 inhibitor ATN-224 drastically reduces the ability of the lung carcinoma cell line A549 to form colony on soft agar [45]. However, inhibition of SOD1 in normal bronchial epithelial cells had no effect [45]. They further reported the unexpected finding that inhibition of SOD1 leads to an increase rather than a decrease in hydrogen peroxide [45]. They found that this increase in hydrogen peroxide resulted from the inhibition of the GPX enzymes by superoxide. Further, their study reported that this elevation in hydrogen peroxide leads to activation of p38 and a decrease in the anti-apoptotic factor MCL1 [45], suggesting that inhibition of SOD1 induces cell death by apoptosis. In light of this study and the fact that SOD1 is expressed in the cytoplasm and the mitochondria, the mechanism by which SOD1 inhibitors cause cell death is likely through a combination of regulated mechanism (apoptosis) and unregulated mechanism (oxidative damage to the organelle).

### SOD1 is overexpressed in breast cancer

Following the finding that SIRT3 is decreased in 87% of breast cancer [46], we hypothesized that decreased expression of SIRT3 may be counterbalanced by an up-regulation of SOD1. As a result, the total level of ROS in the mitochondria is maintained within a window that is compatible with cell survival. We recently reported, using a panel of breast cell lines, that SOD1 is overexpressed while SIRT3 is decreased [44]. Conversely, SOD1 is decreased and SIRT3 elevated in the non-malignant cell line MCF10A. We also reported the same inverse correlation between SIRT3 and SOD1 using immunohistochemistry on a tissue microarray of primary breast cancers [44].

Since the decrease in SIRT3 appears to be a requirement for the Warburg effect and the reprogramming toward glycolysis [46], the increase in SOD1 may act as a general ROS rheostat mechanism. If so, one prediction is that increased level of SOD1 may be independent of the oncogene driving the proliferation of tumors. To further test this possibility, we analyzed the level of SOD1 in MMTV-Wnt, MMTV-erbB2 and MMTV-Myc mouse models of breast cancer. We found that, while the SOD1 protein was undetectable in the mammary ducts of wild type females, high levels of SOD1 protein was detected in all three mammary tumor models [44]. Therefore, our data indicates that the overexpression of SOD1 is a frequent occurrence in breast cancer.

In addition, the analysis of SOD1 in human primary breast cancers revealed that SOD1 accumulates not only in the cytoplasm but also in the nucleus of cancer cells. This finding suggests that the nuclear fraction of SOD1 may also play an important role in the survival of cancer cells. However, the role of the nuclear-fraction of SOD1 remains largely unknown. One possibility arises from the observation that SOD1 binds to the estrogen receptor alpha (ERα) [47]. Importantly, interaction of SOD1 with the ERα is only observed when the receptor is bound to DNA. Further, the binding of SOD1 to the ERα was reported to enhance its transcriptional activity [47].

### Overexpression of SOD1 in the nucleus and the IMS may participate in the induction of the mitochondrial unfolded protein response

The mitochondrial unfolded protein response (UPR^mt^) involves several players and results in different outcomes such as activation of mitochondrial protein-quality control, mitophagy and the mitochondrial anti-oxidant machinery, including SOD2 [48-52]. Ultimately, the result of these outcomes is the protection of the mitochondrial network against the detrimental effect of accumulation of misfolded proteins in the organelle.

**Fig 2:**
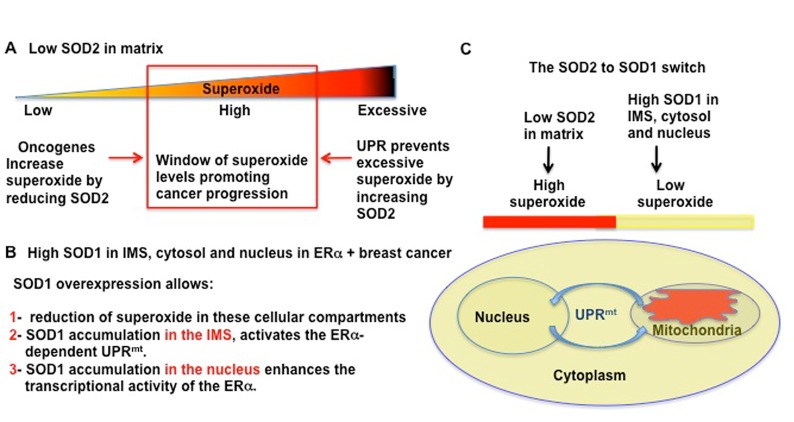
Oncogenes and UPR keep ROS in the high level range A) Illustration of our hypothesis regarding the role of the reduction in SOD2 in the regulation of ROS in breast cancer. We hypothesize that oncogenic activation mediates a direct reduction in SOD2 levels, allowing superoxide level to rise from low levels, as in normal cells, to high levels. The resulting high levels of ROS act to assist the metabolic reprogramming of cancer cells. However, since excessive ROS would cause irreversible damage to the mitochondria, under stress conditions where ROS levels raise further, the UPR^mt^ is activated to elevate SOD2. As a consequence, ROS levels are decreased from excessive to high range. B) List of dismutase-dependent and independent roles of SOD1 in cancer. C) Diagram of how SOD2 to SOD1 switch create high ROS in matrix, but lower ROS in the other cellular compartments, while simultaneously activating the UPR^mt^.

Among the players of the UPR^mt^, we reported that the ERα plays an important role specifically in response to accumulation of proteins in the IMS [52]. Notably, we showed that accumulation of SOD1 in the IMS is a potent activator of the UPR^mt^ [52]. Therefore, it is tempting to speculate that the combined effects of the accumulation of SOD1 in the IMS, to activate the UPR^mt^ and SOD1 in the nucleus, to enhance the activity of the ERα may cooperate to amplify this cytoprotective response (Figure 2).

## CONCLUDING REMARKS

SOD1 is rapidly emerging as a novel target for cancer therapy. The deregulation of the anti-oxidant machinery of the mitochondrial matrix appears to play a critical role during transformation, by leading to elevated ROS in the matrix (Figure 2). In this context, the overexpression of SOD1 in the cytoplasm, the IMS, and the nucleus is likely to act by maintaining low ROS levels in these compartments of the cell (Figure 2). In addition to these dismutase-dependent actions of SOD1, we propose that the activation of the ERα in the nucleus and the ability to activate the UPR^mt^ represent additional dismutase-independent roles of SOD1. Collectively, these dismutase-dependent and independent roles of SOD1 may explain the apparent addiction of cancer cells to this enzyme. Clearly, more studies are required to test these possibilities.
